# Super-Twisting Sliding Mode Trajectory Tracking Control of an Underwater Manipulator Subject to Input Saturation Constraints

**DOI:** 10.3390/s26051607

**Published:** 2026-03-04

**Authors:** Hui Yang, Siyu Niu, Xuyu Shen, Zhenzhong Chu

**Affiliations:** College of Mechanical Engineering, University of Shanghai for Science and Technology, Shanghai 200090, China; 232191454@st.usst.edu.cn (H.Y.); 241190150@st.usst.edu.cn (S.N.); 221170113@st.usst.edu.cn (X.S.)

**Keywords:** underwater manipulator, super-twisting algorithm, extended state observer, input saturation compensation

## Abstract

**Highlights:**

**What are the main findings?**

- A super-twisting sliding mode controller with ESO is proposed for underwater manipulators.
- Input saturation compensation significantly improves tracking robustness.

**What are the implications of the main findings?**

- Robust and accurate trajectory tracking is achieved under actuator saturation.
- Chattering is substantially suppressed without sacrificing robustness.

**Abstract:**

To address the trajectory tracking problem of underwater manipulators operating in complex marine environments with strong multi-degree-of-freedom coupling, pronounced nonlinearities, and actuator saturation constraints, this paper proposes a super-twisting sliding mode control scheme integrated with an extended state observer and an anti-saturation auxiliary system. A dynamic model of the underwater manipulator incorporating major hydrodynamic effects (added mass and drag) is first established. Based on this model, a super-twisting sliding mode controller is designed to achieve fast convergence of the tracking errors while effectively alleviating the chattering phenomenon associated with conventional sliding mode control. An improved extended state observer is then introduced to estimate unmodeled dynamics and external time-varying disturbances in real time, providing feedforward compensation to enhance system robustness. To explicitly handle actuator output limitations, an anti-saturation auxiliary system is further developed to dynamically regulate the control input and mitigate the adverse effects of saturation. Comparative simulation studies conducted on the Oberon7 underwater manipulator demonstrate that the proposed control strategy achieves higher trajectory tracking accuracy, improved disturbance rejection capability, and faster recovery after saturation release compared with conventional control methods. These results indicate that the proposed approach offers an effective and reliable solution for high-precision trajectory tracking control of underwater manipulators under input saturation constraints.

## 1. Introduction

Underwater manipulators serve as essential platforms for deep-sea resource exploration and operations, playing an irreplaceable role in underwater infrastructure maintenance, emergency salvage, and scientific sampling tasks [1,2,3]. However, due to their strongly coupled multi-degree-of-freedom nonlinear dynamics and the influence of complex unstructured environments such as ocean currents and contact collisions, accurate modeling and highly robust control of underwater manipulators remain challenging and have become long-standing research topics in the control community [4]. In particular, underwater operation introduces additional hydrodynamic effects, including added mass, nonlinear fluid damping/drag, and strong coupling forces induced by the surrounding fluid. These effects are commonly described based on marine craft hydrodynamic modeling principles [5], and drag forces are often represented using Morison-type formulations [6]. The presence of such uncertainties significantly increases model complexity and degrades tracking accuracy. Compared with conventional control methods such as proportional–integral–derivative (PID) control [7] and model predictive control (MPC) [8], which are often limited by model accuracy or computational complexity, sliding mode control (SMC) [9] has been widely adopted for underwater manipulator trajectory tracking owing to its inherent robustness and insensitivity to parametric uncertainties and external disturbances.

To further enhance the overall performance of sliding mode control in underwater environments, extensive research efforts have been devoted to its improvement. From the perspective of algorithmic optimization, Zhong et al. [10] and Liu et al. [11] mitigated chattering and enhanced disturbance rejection by introducing adaptive fuzzy gain regulation and improved reaching laws, respectively. Meanwhile, Zaare et al. [12] developed a predefined-time nonsingular terminal sliding mode controller, achieving global convergence within a prescribed time. In terms of environmental adaptability, Mao et al. [13] and Ge et al. [14] validated the effectiveness of sliding mode strategies under complex hydrodynamic conditions by incorporating disturbance observers and optimized reaching laws to address high-speed currents and wave-induced disturbances.

Despite the effectiveness of these improved approaches, the inherent chattering phenomenon of conventional sliding mode control remains a critical factor limiting its achievable precision, which has motivated the development of terminal sliding mode control [15,16] and high-order sliding mode control [17]. As a representative high-order sliding mode technique, super-twisting sliding mode control (STSMC) employs an integral action to generate continuous control signals, thereby substantially mitigating chattering while preserving robustness. The theoretical foundations and finite-time convergence properties of the super-twisting algorithm for the sliding variable have been systematically established within the framework of higher-order sliding modes [18]. In recent years, STSMC has been widely applied in underwater robotic systems. Studies by Liao et al. [19] and Borlaug et al. [20] have demonstrated that incorporating adaptive mechanisms into STSMC can significantly reduce high-frequency chattering and improve position tracking accuracy.

In addition, active disturbance compensation constitutes another effective pathway for improving control performance. By employing disturbance observers (DOBs) [21] or sliding mode observers to estimate model uncertainties and external disturbances and provide feedforward compensation, the burden on the feedback controller can be significantly alleviated [22]. Among various observer designs, the extended state observer (ESO) exhibits notable advantages due to its ability to treat internal uncertainties and external disturbances as a lumped disturbance and estimate them in a unified manner, while relying minimally on an accurate system model. Consequently, ESO has shown great potential in manipulator control applications. For instance, Wang et al. [23,24] combined a finite-time ESO with terminal sliding mode control to achieve fast and stable control performance under complex hydrodynamic conditions.

Although these studies have improved trajectory tracking performance to some extent, several practical issues remain insufficiently addressed. First, most STSMC–ESO-based approaches focus primarily on chattering mitigation and disturbance rejection, while actuator torque saturation—an inevitable constraint in practical underwater manipulators—is rarely incorporated into the control design and stability analysis. Actuator saturation may lead to performance degradation, loss of sliding motion, and delayed recovery after saturation release. From a general control perspective, this problem is closely related to the classical anti-windup framework. Second, the interaction between disturbance estimation, sliding dynamics, and saturation recovery is often treated separately rather than within a unified stability-oriented framework. Therefore, achieving coordinated disturbance rejection, chattering mitigation, and saturation-aware robustness remains a critical challenge. To address these issues, this paper proposes a saturation-aware trajectory tracking control scheme that integrates super-twisting sliding mode control (STSMC) with an extended state observer (ESO) and an auxiliary anti-saturation dynamic system. The main contributions of this work are summarized as follows:

1. An STSMC–ESO composite framework is developed for underwater manipulators, where the ESO estimates lumped disturbances in real time to provide feedforward compensation, thereby reducing reliance on high feedback gains.
2. An auxiliary anti-saturation dynamic system is introduced to explicitly address actuator torque constraints, improving tracking robustness and accelerating recovery after saturation release.
3. A unified Lyapunov-based stability analysis of the overall closed-loop system is provided, demonstrating global exponential asymptotic stability under saturation constraints.

The remainder of this paper is organized as follows. Section 2 presents the dynamic model of the underwater manipulator incorporating hydrodynamic effects and lumped uncertainties. Section 3 describes the design of the anti-saturation auxiliary system, the ESO, and the super-twisting sliding mode controller, followed by the stability analysis. Section 4 provides comparative simulation results and discussion. Finally, Section 5 concludes the paper and outlines future research directions.

## 2. Dynamic Model

Considering the effects of added mass and hydrodynamic drag in the underwater environment, the dynamics of a six-degree-of-freedom underwater manipulator are described based on the Lagrange formulation as

(1) $$M(q)\ddot{q}+C(q,\dot{q})\dot{q}+g(q)=\tau$$

where M(q) is the symmetric positive-definite inertia matrix with $M(q)\in R^{n\times n}$; $\;C(q,\dot{q})$ denotes the Coriolis and centrifugal matrix with $C(q,\dot{q})\in R^{n\times n}$; g(q) is the gravity vector with $g(q)\in R^{n}$; $q\in R^{n}$ represents the joint position vector; and $\tau \in R^{n}$ denotes the joint control torque vector.

In practical engineering applications, due to the difficulty of accurately identifying hydrodynamic coefficients and the influence of complex ocean current environments, the system is subject to parameter perturbations and external disturbances. Therefore, the dynamic model is reformulated into a structure that explicitly includes uncertain terms as

(2) $$(M_{0}(q)+\Delta M(q))\ddot{q}+(C_{0}(q,\dot{q})+\Delta C(q,\dot{q}))\dot{q}+g\prime_{0}(q)+\Delta g\prime (q)+\tau_{f}=\tau +\tau_{dist}+\tau_{i}$$

where $M_{0}(q),C_{0}(q,\dot{q}),g\prime_{0}(q)$ are the nominal parameter matrices;

$\Delta M(q),\Delta C(q,\dot{q}),\Delta g^{\prime }(q)$ denote the corresponding parameter uncertainty matrices;

$g_{0}^{\prime }\left(q\right),\;\Delta g\prime (q)$ represent the equivalent gravity vectors after accounting for buoyancy effects;

$\tau_{dist}$ is the external disturbance torque vector;

$\tau_{f}$ denotes the joint friction torque vector;

$\tau_{i}$ represents the total inertial hydrodynamic torque vector.

Define the lumped disturbance term as

(3) $$T_{d}=-\Delta M\ddot{q}-\Delta C\dot{q}-\Delta G-\tau_{d}-\tau_{f}$$

then the system dynamics can be simplified as

(4) $$M_{0}(q)\ddot{q}+C_{0}(q,\dot{q})\dot{q}+g\prime_{0}(q)=\tau +d$$

Let the state variables of the manipulator system be chosen as

(5) $$x_{1}=q,\;{\;x}_{2}=\dot{q},\;{\;x}_{3}=D=M_{0}^{-1}d$$

then the state-space representation of the underwater manipulator system is given by

(6) $$\left\{\begin{array}{l} {\dot{x}}_{1}=x_{2}\\ {\dot{x}}_{2}=x_{3}+{M_{0}}^{-1}\tau +f\\ {\dot{x}}_{3}=\dot{D} \end{array}\right.$$

where

(7) $$f=-M_{0}{\left(q\right)}^{-1}[C_{0}(q,\dot{q})\dot{q}+g\prime_{0}(q)]$$

## 3. Controller Design

### 3.1. Input Saturation

Due to the physical manufacturing limitations of underwater manipulator actuators, the actual control torque is subject to amplitude constraints. If the controller output exceeds the actuator limits, the resulting saturation-induced truncation error may significantly degrade tracking performance and even lead to closed-loop instability. Considering the input saturation constraint, the system dynamics can be expressed as

(8) $$\left\{\begin{array}{l} {\dot{x}}_{1}=x_{2}\\ {\dot{x}}_{2}=x_{3}+{M_{0}}^{-1}sat(\tau )+f\\ {\dot{x}}_{3}=\dot{D} \end{array}\right.$$

where the saturation function sat(·) is defined as

(9) $$sat(\tau_{i})=\left\{\begin{array}{l} \tau_{i\max},\tau_{i}\geq \tau_{i\max}\\ \tau_{i},\tau_{i\min}<\tau_{i}<\tau_{i\max}\\ \tau_{i\min},\tau_{i}\leq \tau_{i\min} \end{array}\right.$$

in which $\tau_{i}$ denotes the actual control input of the *i*-th joint, $\tau_{imax},\tau_{imin}$ are the upper and lower bounds of the control input, respectively, and sat(·) represents the input saturation function.

To compensate for the adverse effects of input saturation on tracking performance, an auxiliary anti-saturation compensation system is designed as

(10) $$\left\{\begin{array}{l} {\dot{\eta }}_{1}=\eta_{2}-c_{1}\eta_{1}\\ {\dot{\eta }}_{2}=-c_{2}\eta_{2}+{M_{0}}^{-1}\Delta \tau \end{array}\right.$$

where $\eta_{1}\;and\;\eta_{2}$ are internal state variables of the auxiliary system;

$c_{1}=diag(c_{11},c_{12},\cdots c_{1n})$ and $c_{2}=diag\left(c_{21},c_{22},\cdots c_{2n}\right)$ are positive definite design matrices used to regulate the dynamic response of the auxiliary system;

Δτ=sat(τ)−τ denotes the saturation error.

To explicitly address the stability of the auxiliary anti-saturation dynamics in (10), consider the stacked auxiliary state $\eta ={\left[{\eta_{1}}^{T},{\eta_{2}}^{T}\right]}^{T}$. Then (10) can be rewritten in the compact form.

$$\dot{\eta }=A\eta +B\Delta \tau$$

where

$$A=\left[\begin{array}{cc} -c_{1} & I\\ 0 & -c_{2} \end{array}\right],B=\left[\begin{array}{c} 0\\ {M_{0}}^{-1} \end{array}\right]$$

Since $c_{1}$ and $c_{2}$ are diagonal positive definite matrices, the matrix *A* is Hurwitz. Hence, for any given symmetric positive definite matrix *Q* > 0, there exists a unique *P* > 0 satisfying the Lyapunov equation

$$A^{T}P+PA=-Q$$

Choose the Lyapunov candidate

$$V_{\eta }=\eta^{T}P\eta$$

Its time derivative along the trajectories of the auxiliary system yields

$${\dot{V}}_{\eta }=\eta^{T}(A^{T}P+PA)\eta +2\eta^{T}PB\Delta \tau =-\eta^{T}Q\eta +2\eta^{T}PB\Delta \tau$$

To upper-bound the cross term $2\eta^{T}PB\Delta \tau$, define

$$a=Q^{1/2}\eta ,b=Q^{-1/2}PB\Delta \tau$$

which implies $\eta^{T}PB\Delta \tau =a^{T}b$. By Young’s inequality, for any ε>0,

$$2a^{T}b\leq \varepsilon a^{T}a+\frac{1}{\varepsilon }b^{T}b$$

Substituting *a* and *b* back gives

$$2\eta^{T}PB\Delta \tau \leq \varepsilon \eta^{T}Q\eta +\frac{1}{\varepsilon }\Delta \tau^{T}(B^{T}PQ^{-1}PB)\Delta \tau$$

Therefore,

$${\dot{V}}_{\eta }\leq -(1-\varepsilon )\eta^{T}Q\eta +\frac{1}{\varepsilon }\Delta \tau^{T}(B^{T}PQ^{-1}PB)\Delta \tau$$

Define,

$$\kappa ≜\frac{1}{\varepsilon }\lambda_{\max}(B^{T}PQ^{-1}PB)$$

Therefore.

$${\dot{V}}_{\eta }\leq -(1-\varepsilon )\lambda_{\min}(Q)\|\eta {\|}^{2}+\kappa \|\Delta \tau {\|}^{2}$$

By selecting 0<ε<1 (e.g., ε=0.5), the auxiliary system is input-to-state stable (ISS) with respect to the bounded saturation residual Δτ. Consequently, the auxiliary states $\eta_{1}$ and $\eta_{2}$ remain bounded even under severe saturation, and the anti-saturation mechanism does not destabilize the overall closed-loop system.

Let $q_{d}$ be the desired joint trajectory. Assisted by the auxiliary system, a new set of tracking errors is defined as

(11) $$\left\{\begin{array}{l} \sigma_{1}=x_{1}-q_{d}-\eta_{1}\\ \sigma_{2}=x_{2}-{\dot{q}}_{d}-\alpha_{1}-\eta_{2} \end{array}\right.$$

where $\alpha_{1}=-c_{1}(x_{1}-q_{d})$ is a virtual control term introduced to improve tracking performance.

The time derivative of $\sigma_{1}$ is given by

(12) $${\dot{\sigma }}_{1}={\dot{x}}_{1}-{\dot{q}}_{d}-{\dot{\eta }}_{1}$$

Substituting (7) and (8) into (9) yields

(13) $$\begin{array}{ll} {\dot{\sigma }}_{1} & =\sigma_{2}+\alpha_{1}+\eta_{2}-{\dot{\eta }}_{1}\\ & =\sigma_{2}+\alpha_{1}+c_{1}\eta_{1}\\ & =\sigma_{2}+c_{1}(\eta_{1}-x_{1}+q_{d})\\ & =\sigma_{2}-c_{1}\sigma_{1} \end{array}$$

Define the Lyapunov function as

(14) $$V_{1}=\frac{1}{2}{\sigma_{1}}^{T}\sigma_{1}$$

Taking its time derivative gives

(15) $$\begin{array}{ll} {\dot{V}}_{1} & ={\sigma_{1}}^{T}{\dot{\sigma }}_{1}={\sigma_{1}}^{T}(\sigma_{2}-c_{1}\sigma_{1})\\ & ={\sigma_{1}}^{T}\sigma_{2}-{\sigma_{1}}^{T}c_{1}\sigma_{1} \end{array}$$

If $\sigma_{2}=0$, then ${\dot{V}}_{1}\leq 0$, indicating that the first subsystem is stable.

The derivative of $\sigma_{2}$ is obtained as

(16) $$\begin{array}{ll} {\dot{\sigma }}_{2} & ={\dot{x}}_{2}-{\ddot{q}}_{d}-{\dot{\sigma }}_{1}-{\dot{\eta }}_{2}\\ & =f+{M_{0}}^{-1}sat(\tau )+{M_{0}}^{-1}d-{\dot{\sigma }}_{1}+c_{2}\eta_{2}-{M_{0}}^{-1}\Delta \tau -{\ddot{q}}_{d}\\ & =f+{M_{0}}^{-1}\tau +{M_{0}}^{-1}d-{\dot{\sigma }}_{1}+c_{2}\eta_{2}-{\ddot{q}}_{d} \end{array}$$

### 3.2. Observer Design

In this section, an extended state observer (ESO) is employed to simultaneously estimate the system states and the lumped disturbance. Let the estimated states be denoted by $z_{i}(i=1,2,3)$, and define the estimation errors as $\Delta x_{i}=x_{i}-z_{i}$. The ESO is designed as

(17) $$\left\{\begin{array}{l} {\dot{z}}_{1}=z_{2}+l_{1}\Delta x_{1}\\ {\dot{z}}_{2}=z_{3}+l_{2}\Delta x_{1}+{M_{0}}^{-1}sat(\tau )+f\\ {\dot{z}}_{3}=l_{3}\Delta x_{1} \end{array}\right.$$

where $l_{1},l_{2},l_{3}$ are the observer gain matrices. The estimated states $z_{1}$ and $z_{2}$ are not directly used in the feedback loop, but can serve as optional filtered estimates in the presence of measurement noise. In this study, the measured states are used for feedback to avoid unnecessary estimator–controller coupling and to maintain clarity in stability analysis. Therefore, the role of the ESO in this work is primarily disturbance estimation rather than full state replacement.

To simplify parameter tuning while ensuring ESO stability, the observer poles are placed at bandwidth $-\omega_{0}$, yielding

(18) $$\left\{\begin{array}{l} l_{1i}=3\omega_{0i}\\ l_{2i}=3{\omega_{0i}}^{2}\\ l_{3i}={\omega_{0i}}^{2} \end{array}\right.$$

However, a conventional linear ESO suffers from a trade-off between bandwidth and stability: increasing the bandwidth improves disturbance estimation accuracy but may cause instability. Therefore, a time-varying bandwidth is introduced as

(19) $$\omega_{i}=\omega_{li}+(\omega_{ni}-\omega_{li}){\left(\frac{t}{T_{ramp}}\right)}^{2}$$

where $T_{ramp}$ is the total duration of the bandwidth ramping process, and $\omega_{m}\;and\;\omega_{l}$ denote the normal operating bandwidth and the initial low bandwidth, respectively. This design effectively mitigates the initial differentiation peak and improves both disturbance estimation speed and accuracy. The stability proof can be found in [21].

### 3.3. Super-Twisting Sliding Mode Controller Design

**Figure 1:**
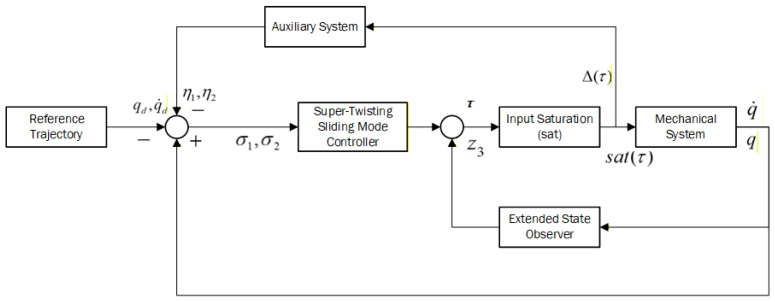
Control flow chart.

The overall control structure is illustrated in Figure 1. Define the sliding surface as

(20) $$s=\lambda \sigma_{1}+\sigma_{2}$$

where λ is the sliding surface gain matrix.

Taking the derivative of (20) yields

(21) $$\begin{array}{ll} \dot{s} & =\lambda {\dot{\sigma }}_{1}+{\dot{\sigma }}_{2}\\ & =\lambda (\sigma_{2}-c_{1}\sigma_{1})+f+{M_{0}}^{-1}\tau +{M_{0}}^{-1}d-{\dot{\sigma }}_{1}+c_{2}\eta_{2}-{\ddot{q}}_{d} \end{array}$$

Unlike conventional sliding mode control, the super-twisting sliding mode controller generates the actual control input through integration and does not involve high-frequency switching terms, thereby substantially mitigating chattering. The super-twisting control law is designed as

(22) $$\left\{\begin{array}{l} \dot{s}=-K_{1}|s{|}^{1/2}\tanh(s/\varepsilon )-ks+z\\ \dot{z}=-K_{2}\tanh(s/\varepsilon ) \end{array}\right.$$

and the final super-twisting sliding mode controller is given by

(23) $$\tau =M_{0}[-\lambda (\sigma_{2}-c_{1}\sigma_{1})-f+{\dot{\sigma }}_{1}-c_{2}\eta_{2}+{\ddot{q}}_{d}-z_{3}-K_{1}|s{|}^{1/2}\tanh(s/\varepsilon )-ks+z]$$

### 3.4. Stability Analysis

Define the Lyapunov function as

(24) $$V_{2}=V_{1}+\frac{1}{2}s^{T}s$$

Taking its derivative yields

(25) $$\begin{array}{ll} {\dot{V}}_{2} & ={\dot{V}}_{1}+s^{T}\dot{s}\\ & ={\sigma_{1}}^{T}\sigma_{2}-{\sigma_{1}}^{T}c_{1}\sigma_{1}+s^{T}[\lambda (\sigma_{2}-c_{1}\sigma_{1})+f+{M_{0}}^{-1}\tau +{M_{0}}^{-1}d-{\dot{\sigma }}_{1}+c_{2}\eta_{2}-{\ddot{q}}_{d}] \end{array}$$

Substituting (23) into (25) gives

(26) $$\begin{array}{ll} {\dot{V}}_{2} & ={\sigma_{1}}^{T}\sigma_{2}-{\sigma_{1}}^{T}c_{1}\sigma_{1}+s^{T}(-K_{1}|s{|}^{1/2}\tanh(s/\varepsilon )-ks+z+{M_{0}}^{-1}d-z_{3})\\ & ={\sigma_{1}}^{T}\sigma_{2}-{\sigma_{1}}^{T}c_{1}\sigma_{1}-ks^{T}s-K_{1}s^{T}|s{|}^{1/2}\tanh(s/\varepsilon )-K_{2}s^{T}\int \tanh(s/\varepsilon )dt\\ & +s^{T}({M_{0}}^{-1}d-z_{3}) \end{array}$$

Let $\tilde{d}=M_{0}^{-1}d-z_{3}$. From the observer dynamics, $\tilde{d}$→0, implying boundedness. Incorporating this term into the integral yields

(27) $$\begin{array}{ll} {\dot{V}}_{2} & \leq {\sigma_{1}}^{T}\sigma_{2}-{\sigma_{1}}^{T}c_{1}\sigma_{1}-ks^{T}s-K_{1}|s||s{|}^{1/2}-|s|\int (K_{2}-\dot{\tilde{d}})dt\\ & \leq {\sigma_{1}}^{T}\sigma_{2}-{\sigma_{1}}^{T}c_{1}\sigma_{1}-ks^{T}s-K_{1i}|s_{i}||s_{i}{|}^{1/2}-|s_{i}|\int (K_{2i}-{\dot{\tilde{d}}}_{i})dt \end{array}$$

By choosing $K_{2}$ such that $K_{2i}\geq {\dot{\tilde{d}}}_{i}$, (27) can be simplified as

(28) $${\dot{V}}_{2}\leq {\sigma_{1}}^{T}\sigma_{2}-{\sigma_{1}}^{T}c_{1}\sigma_{1}-ks^{T}s$$

Since the gains are assigned independently for each joint (i.e., diagonal structure), the quadratic terms in ${\dot{V}}_{2}$ are separable across joints. Hence,

$${\dot{V}}_{2}\leq \sum_{i=1}^{n}\left(\sigma_{1i}\sigma_{2i}-c_{1i}{\sigma_{1i}}^{2}-k_{i}{s_{i}}^{2}\right)$$

Define the stacked error vector

$$\bar{\sigma }=\left[\begin{array}{l} \sigma_{1}\\ \sigma_{2} \end{array}\right]\in {\mathbb{R}}^{2n}$$

For each joint i, define the local vector

$${\bar{\sigma }}_{i}=\left[\begin{array}{l} \sigma_{1i}\\ \sigma_{2i} \end{array}\right]$$

and construct the symmetric matrix

$$Q_{i}=\left[\begin{array}{cc} c_{1i}+k_{i}{\lambda_{i}}^{2} & k_{i}\lambda_{i}-\frac{1}{2}\\ k_{i}\lambda_{i}-\frac{1}{2} & k_{i} \end{array}\right]$$

Stacking all joints yields the block-diagonal matrix

$$Q=diag(Q_{1},\ldots ,Q_{n})\in {\mathbb{R}}^{2n\times 2n}$$

Using $s=\Lambda \sigma_{1}+\sigma_{2}$, one obtains for each joint

$${{\bar{\sigma }}_{i}}^{T}Q_{i}{\bar{\sigma }}_{i}=c_{1i}{\sigma_{1i}}^{2}-\sigma_{1i}\sigma_{2i}+k_{i}{s_{i}}^{2}$$

Therefore,

$${\bar{\sigma }}^{T}Q\bar{\sigma }={\sigma_{1}}^{T}c_{1}\sigma_{1}-{\sigma_{1}}^{T}\sigma_{2}+s^{T}ks$$

Substituting (30) into (28) yields

$${\dot{V}}_{2}\leq -{\bar{\sigma }}^{T}Q\bar{\sigma }$$

Since each $Q_{i}$ is symmetric, its positive definiteness can be verified using Sylvester’s criterion for 2×2 matrices.

For each joint i, the leading principal minors are

$$\Delta_{1i}=q_{11,i}=c_{1i}+k_{i}{\lambda_{i}}^{2}$$

$$\Delta_{2i}=\det(Q_{i})=(c_{1i}+k_{i}{\lambda_{i}}^{2})k_{i}-{\left(k_{i}\lambda_{i}-\frac{1}{2}\right)}^{2}$$

Expanding the equation above,

$$\begin{array}{ll} \det(Q_{i}) & =k_{i}c_{1i}+{k_{i}}^{2}{\lambda_{i}}^{2}-({k_{i}}^{2}{\lambda_{i}}^{2}-k_{i}\lambda_{i}+\frac{1}{4})\\ & =k_{i}(c_{1i}+\lambda_{i})-\frac{1}{4} \end{array}$$

Therefore, $Q_{i}>0$ holds if

$$k_{i}>0,c_{1i}+\lambda_{i}>\frac{1}{4k_{i}},\forall i.$$

Since $Q=diag(Q_{1},\ldots ,Q_{n})$ is block-diagonal, it follows that

$$Q>0\Leftrightarrow Q_{i}>0,\forall i.$$

Hence,

$${\dot{V}}_{2}\leq -{\bar{\sigma }}^{T}Q\bar{\sigma }\leq 0$$

which guarantees global exponential asymptotic stability of the closed-loop system.

$$Q=\left[\begin{array}{cc} c_{1}+\lambda^{T}k\lambda & k\lambda -\frac{1}{2}E\\ k\lambda -\frac{1}{2}E & k \end{array}\right]$$

$$\sigma^{T}Q\sigma ={\sigma_{1}}^{T}c_{1}\sigma_{1}-{\sigma_{1}}^{T}\sigma_{2}+ks^{T}s$$

$${\dot{V}}_{2}\leq -\sigma^{T}Q\sigma \leq 0$$

$$|Q|=k(c_{1}+k\lambda^{2})-{\left(k\lambda -\frac{1}{2}\right)}^{2}=k(c_{1}+\lambda )-\frac{1}{4}$$

## 4. Simulation Comparisons and Results Analysis

**Figure 2:**
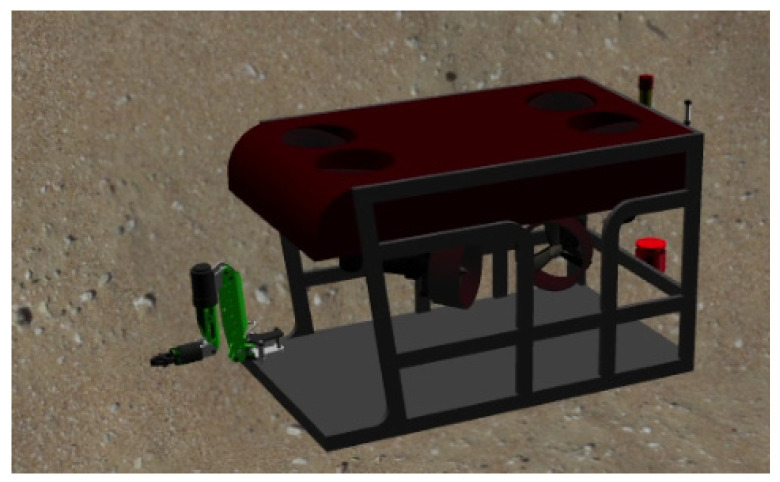
Simulation environment loading.

To validate the effectiveness of the proposed super-twisting sliding mode control (STSMC) strategy with input saturation constraints combined with an extended state observer (ESO), a six-degree-of-freedom underwater manipulator system, Oberon7, is selected as the research object. Numerical simulations are conducted in Gazebo 11.10.2 (Open Source Robotics Foundation) under Ubuntu 20.04, as illustrated in Figure 2.

The model parameters of Oberon7 include link masses, center-of-mass positions, and inertia tensors, which are listed in Table 1. The motion ranges and maximum angular velocity limits of each joint are given in Table 2. To ensure that the generated reference trajectories satisfy the physical constraints of the actuators, the amplitude and frequency parameters of the sinusoidal trajectories are individually designed for each joint, such that the resulting trajectories do not exceed the prescribed maximum velocity limits throughout the entire motion.

Table 1 presents the detailed model parameters of the Oberon7 manipulator, including the mass, center-of-mass position, and inertia tensor of each link.

Table 2 summarizes the allowable joint motion ranges and maximum angular velocities.

Controller Configurations

For performance comparison, three control schemes are considered:

Conventional sliding mode control (SMC);
Super-twisting sliding mode control (STSMC);
STSMC combined with an extended state observer (STSMC + ESO).

The corresponding control laws are given as follows:

(29) $$\tau =M_{0}[{\ddot{q}}_{d}+\Lambda \dot{e}+ks+sign(s)]+C_{0}\dot{q}+g_{0}+f$$

(30) $$\tau =M_{0}[-\lambda (\sigma_{2}-c_{1}\sigma_{1})-f+{\dot{\sigma }}_{1}-c_{2}\eta_{2}+{\ddot{q}}_{d}-K_{1}|s{|}^{1/2}\tanh(s/\varepsilon )-ks+z]$$

(31) $$\tau =M_{0}[-\lambda (\sigma_{2}-c_{1}\sigma_{1})-f+{\dot{\sigma }}_{1}-c_{2}\eta_{2}+{\ddot{q}}_{d}-z_{3}-K_{1}|s{|}^{1/2}\tanh(s/\varepsilon )-ks+z]$$

To limit the length of this section, only the tracking results of the first three key joints are presented, since the remaining joints exhibit similar response characteristics. The controller parameters for these three joints are listed in Table 3.

The controller parameters in Table 3 are determined following a structured bandwidth-based and time-scale separation design procedure.

First, the sliding surface gain *λ* is selected to ensure that the closed-loop error dynamics are faster than the dominant reference frequency. Accordingly, progressively larger values (4, 6, and 10) are adopted for Joint1–Joint3, providing faster convergence for joints with stronger coupling and higher disturbance sensitivity.

Second, the SMC switching gain *k* is chosen slightly above the estimated upper bound of the equivalent disturbance magnitude. The larger value for Joint2 (28) reflects its higher disturbance level, while moderate values (18 and 20) are sufficient for the remaining joints.

Third, the STSMC gains $K_{1}$ and $K_{2}$ are determined according to the estimated upper bound of the disturbance to ensure rapid convergence of the sliding variable. The gains increase from Joint1 (2, 1) to Joint3 (12, 8), consistent with the increasing disturbance variation rate and coupling intensity.

Fourth, the ESO bandwidth $\omega_{n}$ is selected to be on the same order as, or moderately higher than, the closed-loop bandwidth implied by the sliding surface gain ($\omega_{n}\approx 1∼2\;\lambda$), while avoiding excessive noise amplification. Thus, larger bandwidths (10, 10) are assigned to Joint2 and Joint3, whereas a moderate value (5) is used for Joint1.

Finally, the anti-saturation auxiliary gains $c_{1}$ and $c_{2}$ are chosen according to time-scale separation principles such that the saturation recovery dynamics are faster than the nominal closed-loop dynamics. For the joint with higher torque demand (Joint3), larger gains (15, 15) are adopted to accelerate saturation recovery, while moderate gains (1, 1) are sufficient for Joint1 and Joint2.

Reference Trajectory Design

To ensure that the reference trajectories satisfy amplitude and velocity constraints while maintaining sufficient smoothness, sinusoidal functions are adopted to generate the desired joint trajectories:

(32) $$\left\{\begin{array}{l} q_{d}(t)=q_{mid}+A\sin(2\pi ft+\varphi )\\ {\dot{q}}_{d}(t)=A\cdot 2\pi f\cdot \cos(2\pi ft+\varphi )\\ {\ddot{q}}_{d}(t)=-A\cdot {\left(2\pi f\right)}^{2}\cdot \sin(2\pi ft+\varphi ) \end{array}\right.$$

where $Q_{d}(t)$, ${\tilde{q}}_{d}(t),\;and\;{\tilde{q}}_{d}(t)$ denote the desired joint position, velocity, and acceleration, respectively. The midpoint of the joint motion range is defined as

(33) $$q_{mid}=\frac{q_{upper}+q_{lower}}{2}$$

and the trajectory amplitude is

(34) $$A=\frac{q_{upper}-q_{lower}}{2}\cdot \alpha$$

where α∈(0,1) is a scaling factor, chosen as α = 0.8 in this study. The trajectory frequency is determined by

(35) $$f=\frac{v_{\max}}{2\pi A}$$

to ensure that the desired velocity does not exceed the specified maximum joint velocity. The phase angle is given by

(36) $$\varphi =\arcsin(\frac{q_{0}-q_{mid}}{A})$$

which guarantees that the trajectory satisfies the initial condition $q_{d}(0)=q_{0}$.

This trajectory design not only satisfies physical constraints but also provides sufficient smoothness and trackability, making it well suited for sliding mode control performance evaluation.

Disturbance Injection

To evaluate the robustness of the proposed controller against modeling inaccuracies and unmodeled dynamics, a sensitivity analysis is conducted. Specifically, key hydrodynamic parameters (including added mass and drag coefficients) are perturbed by ±30% to emulate modeling errors and environmental variability. In addition, external time-varying disturbance terms are injected into the system to simulate non-stationary environmental effects. It should be emphasized that the simulated UVMS operates in a Gazebo underwater environment where hydrodynamic effects and ambient current disturbances are already included through the underwater simulation plugins (e.g., buoyancy, added mass, hydrodynamic damping, and current-induced loads). Therefore, the closed-loop system is continuously subject to environmental disturbances during the entire simulation. In addition to these environment-induced disturbances, we superimpose an explicit sinusoidal disturbance term to provide a repeatable and controlled benchmark for fair comparison among different control strategies. External disturbances are introduced as follows:

(37) $$\left\{\begin{array}{l} d_{1}=5\sin(0.05\pi t)(10\leq t\leq 20)\\ d_{2}=10\sin(0.05\pi t)(10\leq t\leq 20)\\ d_{3}=30\sin(0.2\pi t)(10\leq t\leq 20) \end{array}\right.$$

To evaluate tracking accuracy, disturbance rejection, and control smoothness, a set of standard time-domain indices is reported for each joint and each controller. We compute the root-mean-square error (RMSE) and ITAE $=\int_{0}^{T}t|e(t)|dt$. To quantify control effort, we report the RMSE of torque. Moreover, to objectively characterize chattering/high-frequency oscillations, we introduce two additional indicators: (i) $RMS(\dot{\tau })$ computed from the numerical derivative of τ(t), and (ii) the high-frequency energy ratio $R_{HF}$, defined as the ratio of spectral energy of τ(t) above a cutoff frequency $f_{c}$ to the total spectral energy. In this study, $f_{c}=10\;Hz$ is used. All indices are computed from the logged trajectories using a unified evaluation script to ensure a fair comparison. Table 4, Table 5 and Table 6 report the above metrics for the azimuth, shoulder, and elbow joints, respectively.

**Elbow Joint Performance Analysis**

**Figure 3:**
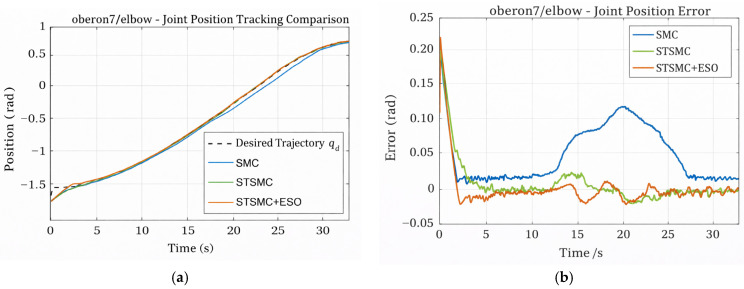
Elbow joint tracking performance: (**a**) position tracking; (**b**) tracking error.

Figure 3a,b present the position responses and tracking errors of the elbow joint under the three control schemes. All controllers are capable of tracking the reference trajectory; however, notable performance differences can be observed. During periods of strong disturbances (e.g., t = 15–20 s), conventional SMC exhibits pronounced error fluctuations and inferior steady-state accuracy, with a maximum tracking error of 0.19848 rad and an RMS error of 0.06638 rad.

In contrast, STSMC significantly improves tracking accuracy, reducing the RMS error to 0.03399 rad. When ESO is further incorporated, the STSMC + ESO scheme achieves the best performance, with the RMS error further reduced to 0.0202 rad and the maximum error constrained within 0.21921 rad. Moreover, the error curve remains smooth throughout the entire process, clearly demonstrating the effectiveness of disturbance observation in enhancing tracking accuracy and robustness.

**Figure 4:**
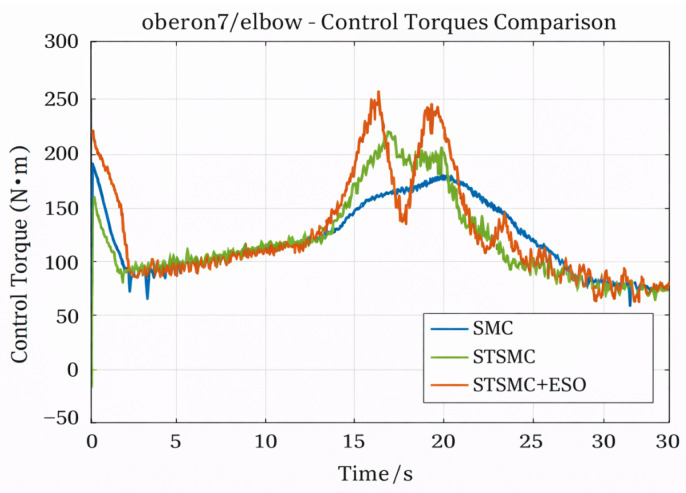
Elbow control torque comparison chart.

Figure 4 compares the control torque outputs. Conventional SMC exhibits abrupt torque variations around t = 3 s, with a peak value of 193.05 N·m, which may induce actuator shocks. In contrast, STSMC and STSMC + ESO generate significantly smoother control inputs, with peak torques limited to 221.79 N·m and 257.04 N·m, respectively, both remaining within saturation limits. Although STSMC + ESO produces a slightly higher RMS torque (139.23 N·m) and increased control energy consumption (∫|τ|dt = 3895.40 N·m·s), it responds more rapidly to disturbances and exhibits better matching between control effort and disturbance compensation, indicating stronger adaptive and dynamic compensation capabilities.

**Figure 5:**
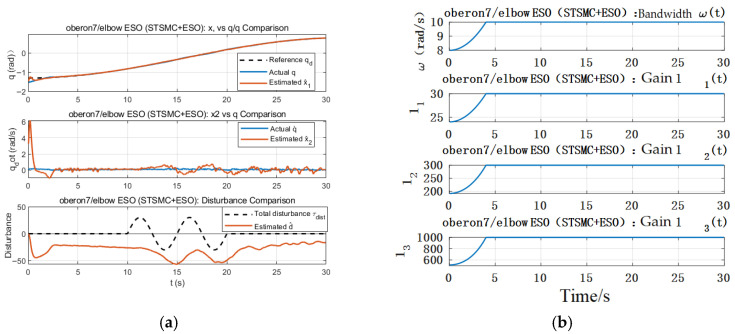
Elbow ESO estimated performance and parameter dynamic variation graph. (**a**) ESO Estimated Performance (Position and Velocity). (**b**) ESO Bandwidth and Observer Gains Evolution.

Figure 5a illustrates the ESO estimation performance, where ${\hat{x}}_{1}\;and\;{\hat{x}}_{2}$ denote the estimated position and velocity, and $\hat{d}$ represents the estimated disturbance. The results show that the ESO achieves rapid convergence of state estimates, and the disturbance estimate closely follows the trend of the external disturbance $\tau_{\mathrm{dist}}$ after approximately t ≈ 3 s, confirming the observer’s real-time performance and estimation accuracy. It is important to clarify that the lumped disturbance term includes not only the external disturbance input, but also unmodeled dynamics, parametric uncertainties, hydrodynamic coupling effects, friction, and modeling errors. Therefore, a perfect match between the injected disturbance and the ESO estimation is not generally expected. Figure 5b shows the dynamic evolution of the ESO bandwidth and observer gains, where $\omega ,l_{1},l_{2},l_{3}$ reach their nominal operating values at approximately t ≈ 4 s. The adaptive bandwidth design effectively mitigates the initial differentiation peak problem commonly encountered in linear ESO structures.

**Figure 6:**
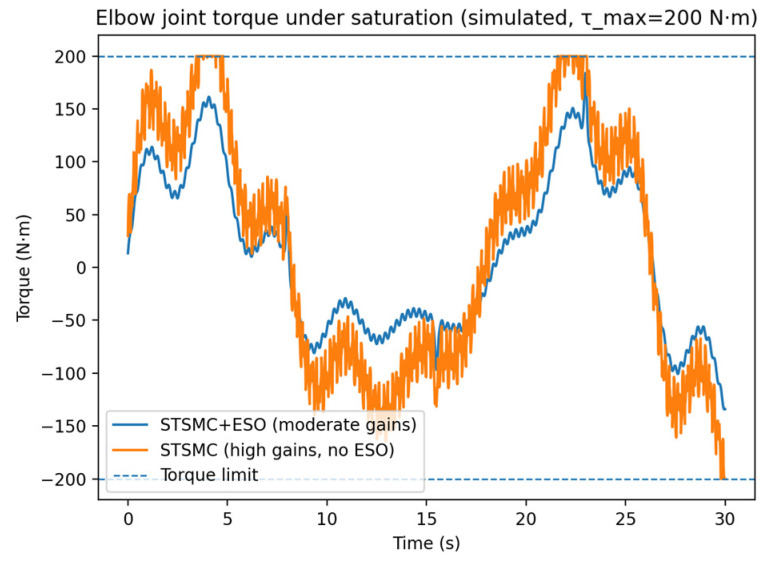
Elbow joint torque under saturation.

To clarify the practical advantage of ESO, an ablation study is conducted on the elbow joint by comparing: (i) the proposed STSMC + ESO with moderate feedback gains, and (ii) a high-gain STSMC without ESO, whose gains are increased to compensate for disturbances under the same torque saturation limit $\tau_{max}=200\;N\cdot m$. The results are summarized in Figure 6 and Table 7.

As shown in Table 7, STSMC + ESO achieves smaller RMS/peak tracking errors than the high-gain STSMC baseline. More importantly, the high-gain STSMC (without ESO) exhibits a substantially larger torque variation index (RMS $\left(d\tau /dt\right)$) and a higher saturation time ratio, indicating more severe chattering and more frequent saturation exposure.

These results demonstrate that the ESO provides active lumped-disturbance compensation in a feedforward manner, which reduces the required feedback gain margin for achieving similar tracking performance. Consequently, the proposed STSMC + ESO yields smoother control effort, mitigates saturation, and improves recovery behavior under disturbance transients.

To evaluate the influence of the ESO bandwidth parameter, a sensitivity study is conducted by varying $\omega_{n}$ among 5, 10, and 15 while keeping other parameters unchanged. As shown in Table 8, increasing the bandwidth from 5 to 10 significantly improves tracking performance, reducing RMSE by approximately 13.5% and ITAE by 24.1%. However, further increasing the bandwidth to 15 yields only a marginal improvement in tracking accuracy while increasing the high-frequency energy ratio $R_{HF}$, indicating enhanced noise amplification. Therefore, $\omega_{n}=10$ is selected as a compromise between estimation speed and robustness.

**Shoulder Joint Performance Analysis**

**Figure 7:**
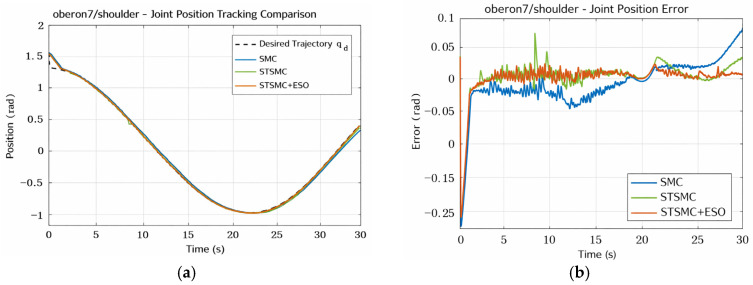
Shoulder joint position tracking and error comparison chart. (**a**) Position Tracking for Shoulder Joint. (**b**) Tracking Error for Shoulder Joint.

**Figure 8:**
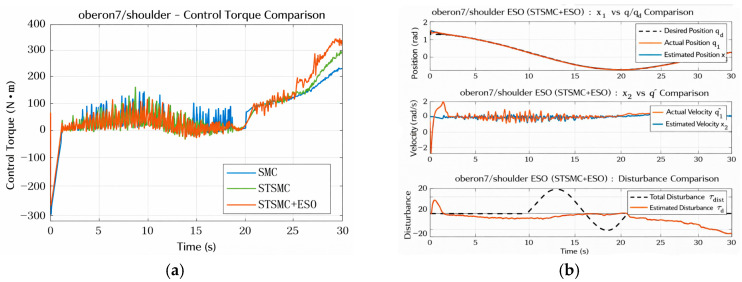
Shoulder control torque and ESO estimation performance. (**a**) Control Torque Comparison for Shoulder Joint. (**b**) ESO Estimation Performance for Shoulder Joint.

The comparative results of the three control schemes for the Shoulder joint are shown in Figure 7 and Figure 8. As depicted in Figure 7a, all approaches are capable of tracking the desired trajectory with generally satisfactory performance, achieving high-precision trajectory regulation overall. The position tracking errors are compared in Figure 7b. It can be observed that the conventional SMC exhibits a slight divergence in the mid-to-late stage, with a maximum error of 0.24544 rad and an RMS error of 0.04426 rad, indicating relatively large overall deviations. After introducing STSMC, the error is significantly reduced, with the RMS error decreasing to 0.03250 rad. With further incorporation of the ESO compensation mechanism, the error convergence is enhanced, and the RMS error is further reduced to 0.02956 rad, accompanied by smaller error fluctuations and noticeably improved tracking stability.

Figure 8a illustrates the variation in the control input torques. Due to the heavy load and strong coupling of the Shoulder joint with other joints, all three controllers generate control signals with relatively large magnitudes. The maximum output torque of SMC reaches 241.62 N·m, while STSMC and STSMC + ESO reach 293.63 N·m and 343.58 N·m, respectively. Although the latter exhibits higher control energy consumption (the integral control effort is 2640.72 N·m·s), it achieves superior tracking accuracy and faster recovery capability, indicating a favorable trade-off between control cost and performance. Compared with SMC (0.0056), STSMC reduces $R_{HF}$ to 0.0021 (62% reduction), and STSMC + ESO further reduces it to 0.0014 (75% reduction), indicating a significant attenuation of high-frequency oscillations.

Figure 8b presents the ESO estimation capability for the system states and the lumped disturbance. The estimated position and velocity closely match the true values, demonstrating the effectiveness of the ESO in state observation. Regarding disturbance estimation, due to complex nonlinear coupling and parametric uncertainties affecting the Shoulder joint, the ESO estimate exhibits a certain deviation from the reference disturbance.

It is worth noting that the disturbance estimation error of the Shoulder joint is noticeably larger than that of the other joints. This phenomenon can be mainly attributed to two factors. First, as a proximal joint with higher equivalent inertia and stronger nonlinear coupling, the Shoulder joint is subject to more complex configuration-dependent dynamics, which makes the lumped disturbance inherently more difficult to approximate through a single matched disturbance channel. Second, the Shoulder joint operates under higher torque demand and may approach saturation limits more frequently, while sampling and noise constraints limit the achievable observer bandwidth. These factors introduce additional discrepancy between the nominal observer model and the actual plant dynamics, resulting in a noticeable estimation bias.

It should be emphasized that the ESO estimation does not aim to reproduce the injected disturbance waveform exactly, but to provide a real-time compensation signal for the lumped disturbance. Therefore, the effectiveness of the ESO is evaluated primarily based on closed-loop tracking performance and robustness rather than pointwise estimation accuracy.

**Azimuth Joint Performance Analysis**

**Figure 9:**
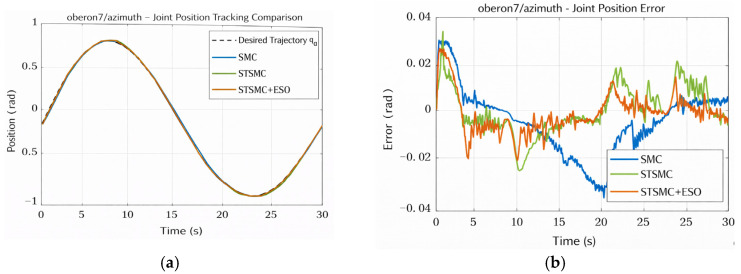
Azimuth joint position tracking and error comparison chart. (**a**) Position Tracking for Azimuth Joint. (**b**) Tracking Error for Azimuth Joint.

**Figure 10:**
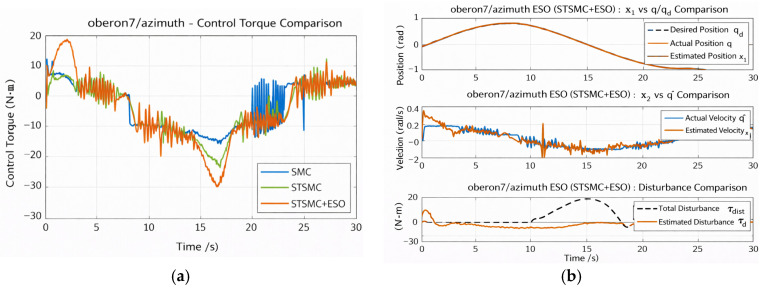
Azimuth control torque and ESO estimation performance. (**a**) Control Torque Comparison for Azimuth Joint. (**b**) ESO Estimation Performance for Azimuth Joint.

The comparative results for the Azimuth joint are shown in Figure 9 and Figure 10. Figure 9a presents the position tracking curves under the three controllers, where all schemes achieve satisfactory tracking performance. Nevertheless, noticeable differences arise in tracking accuracy during disturbance intervals. The corresponding tracking errors are compared in Figure 9b. The STSMC scheme exhibits faster error convergence, with the maximum error decreasing from 0.03871 rad to 0.03658 rad, and the RMS error decreasing from 0.01586 rad to 0.01054 rad. By further introducing the ESO, the system’s robustness against disturbances is strengthened via state observation and compensation, leading to an RMS error of 0.00915 rad and a reduced maximum error of 0.03026 rad, achieving the best tracking performance among the three methods.

Figure 10a compares the control input responses. Within t∈[19, 21] s, $RMS(\dot{\tau })$ reaches 120.984 N·m/s and the high-frequency energy ratio $R_{HF}(f_{c}=10\;Hz)$ increases to 0.04299, which are respectively 9.02× and 49.40× larger than those in the preceding interval. This confirms the localized chattering behavior of SMC. In contrast, STSMC and STSMC + ESO significantly reduce high-frequency torque activity, resulting in smoother control inputs. Although its RMS torque is slightly higher (9.53 N·m) and the accumulated control energy is 241.36 N·m·s (slightly higher than SMC at 200.25 N·m·s), it provides a clear cost–performance advantage in terms of improved tracking accuracy.

Figure 10b further demonstrates the ESO’s estimation capability for the Azimuth joint. The estimates of position, velocity, and the lumped disturbance are highly consistent with the true values, indicating that the ESO effectively captures unmodeled dynamics and external disturbances, thereby providing reliable support for feedforward compensation.

**Validation of the Input Saturation Compensation Mechanism**

**Figure 11:**
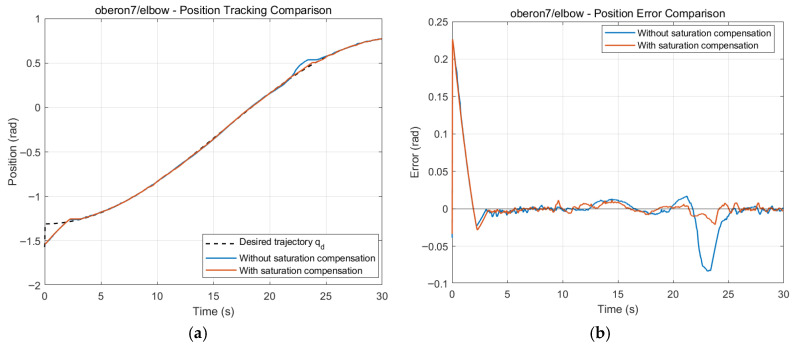
Elbow joint position tracking and error comparison chart. (**a**) Position Tracking for Elbow Joint (with and without Compensation). (**b**)Tracking Error for Elbow Joint (with and without Compensation).

To verify the effectiveness of the input saturation compensation mechanism under actuator-limited conditions, the elbow joint is selected as a representative case. The maximum torque limit is set to 200 N·m, and the performance of the STSMC + ESO controller without and with saturation compensation is compared. The simulation results are shown in Figure 10 and Figure 11.

Figure 11a,b depict the position tracking results and the corresponding tracking errors, respectively. Throughout the overall tracking process, both schemes can largely follow the desired trajectory; however, a clear divergence occurs around t ≈ 24 s, which corresponds to the phase when the control torque transitions from saturation back to the normal operating range. Without compensation, the controller fails to promptly re-establish effective control after the torque recovers, leading to a pronounced short-term trajectory deviation, with a maximum negative error reaching −0.08 rad. In contrast, with the saturation compensation mechanism, the RMS tracking error is reduced from 0.03726 rad to 0.03242 rad, and the controller can rapidly re-enter an effective control region after saturation is released, thereby suppressing the error quickly and restoring the system state.

**Figure 12:**
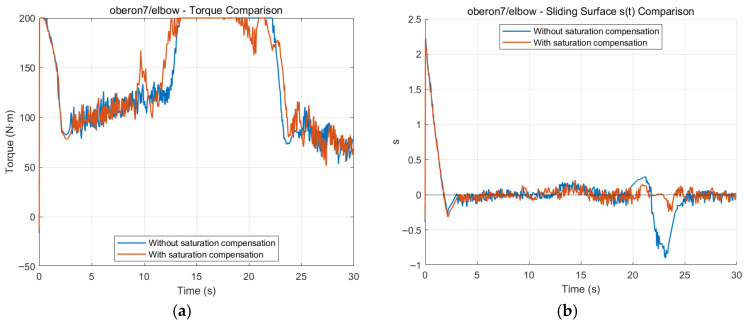
Elbow control torque and sliding mode comparison. (**a**) Control Torque Comparison for Elbow Joint. (**b**) Sliding Mode Dynamics for Elbow Joint.

Figure 12a compares the control torques. Under external disturbances, both schemes generate torque outputs approaching saturation. In the uncompensated case, once the torque reaches the upper bound (200 N·m), the control input cannot be adjusted in time. The recovery time $T_{rec}$ is defined as the time required for the tracking error to re-enter and remain within a prescribed band (∣e(t)∣<0.01 rad for 0.5 s) after saturation is released. The anti-saturation scheme reduces the time spent in full saturation from 9.58 s to 7.35 s and shortens the post-saturation recovery time from 2.754 s to 0.274 s, corresponding to a 90.05% reduction in $T_{rec}$. Figure 12b shows the dynamic evolution of the sliding variable s(t). In the uncompensated case, an evident “sliding-mode escape” occurs near 24 s, i.e., the system state temporarily departs from the sliding surface, which manifests as degradation or loss of sliding-mode effectiveness. In contrast, with saturation compensation, s(t) remains bounded with stable oscillations, and the control loop remains effective, demonstrating that the compensation mechanism not only improves error convergence but also enhances sliding-surface maintenance.

In summary, the proposed saturation compensation mechanism can prevent loss of control when the control output approaches the actuator limit. More importantly, it enables rapid restoration of effective control after saturation is released, shortens the transient error recovery process, and prevents error accumulation and trajectory drift caused by hysteresis effects. This property is particularly important for complex underwater operations involving frequent disturbances and large-amplitude maneuvers, highlighting the practical adaptability and safety advantages of the proposed control strategy.

In principle, the proposed anti-saturation mechanism is joint-independent and can be directly applied to all joints. In this work, the elbow joint is selected as a representative case for clarity and brevity.

**Remarks on Comparisons and Computational Complexity**

In this study, the proposed controller is compared with conventional SMC and STSMC, since these methods share the same sliding-mode framework and provide a fair baseline for evaluating the contributions of the ESO compensation and the anti-saturation auxiliary dynamics.

It is worth noting that other advanced approaches have also been reported for underwater manipulator control, such as adaptive fuzzy sliding mode control [10], Gaussian-process-assisted MPC [8], and prescribed performance control [15]. These methods can achieve high tracking accuracy, but typically require either online optimization (MPC), complex rule-based design and tuning (fuzzy-based control), or careful selection of performance functions and parameters (prescribed performance control). Therefore, implementing and tuning these heterogeneous controllers in a fully fair manner is nontrivial and is beyond the scope of this work.

From the perspective of real-time computation, the proposed method remains computationally lightweight. The ESO consists of linear state updates with a time-varying bandwidth, the super-twisting law involves only algebraic operations and integration, and the anti-saturation auxiliary system is a low-order linear dynamic system. Therefore, the proposed controller does not introduce an optimization loop and is suitable for real-time implementation on embedded robotic platforms.

## 5. Conclusions

This paper addresses the trajectory tracking control problem of underwater manipulators subject to strong multi-degree-of-freedom coupling dynamics, complex external disturbances, and actuator input saturation constraints. A composite control strategy integrating an extended state observer (ESO) with super-twisting sliding mode control (STSMC) is proposed. First, by replacing the conventional sliding mode control law with the super-twisting algorithm, continuity of the control input is achieved, which effectively suppresses system chattering while achieving fast convergence of the tracking errors. On this basis, an extended state observer is employed to estimate unmodeled dynamics and external environmental disturbances in real time and provide feedforward compensation, significantly reducing the controller’s dependence on precise system modeling and enhancing overall system robustness.

To further account for actuator physical limitations in practical engineering applications, an input saturation compensation mechanism is incorporated, ensuring both tracking accuracy and closed-loop stability under torque-constrained conditions. Simulation experiments conducted on the Oberon7 underwater manipulator verify the effectiveness of the proposed control strategy. The results demonstrate that, in terms of root-mean-square tracking error, disturbance recovery speed, and control smoothness, the proposed method significantly outperforms conventional sliding mode control and single super-twisting sliding mode control approaches, while guaranteeing that the control torque remains within physically permissible limits at all times.

In summary, the proposed control scheme exhibits excellent adaptability and practicality in complex underwater environments, providing reliable theoretical support for underwater operational tasks. Future research will focus on the development of adaptive tuning mechanisms for observer parameters and cooperative control strategies for multi-manipulator systems.

## Figures and Tables

**Table 1:** Model Parameters.

Connecting Rod	Mass	Location of the Center of Mass	Inertia Tensor
Azimuth	6.721	[0.0397, 0.0054, 0.0174]	Diag{0.0760, 0.0916, 0.0371}
Shoulder	12.442	[0.3125, 0.0175, 0.0000]	Diag{0.0364, 0.3968, 0.4005}
Elbow	10.312	[0.1252, −0.0746, 0.0000]	Diag{0.0339, 0.0723, 0.0650}
Roll	8.544	[0.0000, 0.0000, 0.1580]	Diag{0.0961, 0.0961, 0.0326}
Pitch	3.902	[0.1040, 0.0033, 0.0000]	Diag{0.0104, 0.0211, 0.0205}
Wrist	3.461	[0.0244, 0.0000, 0.0000]	Diag{0.0077, 0.0053, 0.0055}

**Table 2:** Range of motion and maximum speed limits for each joint.

Joint Names	Range of Motion (°)	Maximum Angular Velocity (rad/s)
Joint1	[−60, 60]	0.17
Joint2	[−60, 90]	0.17
Joint3	[−90, 60]	0.13
Joint4	[−135, 135]	0.085
Joint5	[−90, 90]	0.26
Joint6	[−135, 135]	0.25

**Table 3:** Controller Parameters.

Joint Name	k	λ	$K_{1}$	$K_{2}$	$c_{1}$	$c_{2}$	$\omega_{n}$
Joint1	18	4	2	1	1	1	5
Joint2	28	6	5	3	1	1	10
Joint3	20	10	12	8	15	15	10

**Table 4:** Quantitative performance comparison for the Azimuth joint.

Controller	RMSE (e) (rad)	ITAE (rad·s^2^)	RMSE (τ) (N·m)	$R_{HF}$ (f ≥ 10 Hz)	RMS (dτ/dt) (N·m/s)
SMC	0.0158	4.7628	6.965	0.0039	39.918
STSMC	0.0105	3.4544	7.557	0.0004	29.118
STSMC + ESO	0.0091	2.0738	9.529	0.0003	33.998

**Table 5:** Quantitative performance comparison for the Shoulder joint.

Controller	RMSE (e) (rad)	ITAE (rad·s^2^)	RMSE (τ) (N·m)	$R_{HF}$ (f ≥ 10 Hz)	RMS (dτ/dt) (N·m/s)
SMC	0.0443	12.8666	99.276	0.0011	487.451
STSMC	0.0325	5.9785	108.432	0.0014	504.617
STSMC + ESO	0.0295	3.8701	125.978	0.0023	517.143

**Table 6:** Quantitative performance comparison for the elbow joint.

Controller	RMSE (e) (rad)	ITAE (rad·s^2^)	RMSE (τ) (N·m)	$R_{HF}$ (f ≥ 10 Hz)	RMS (dτ/dt) (N·m/s)
SMC	0.0664	25.7145	126.29	0.0005	106.635
STSMC	0.0339	3.1651	128.781	0.0015	157.424
STSMC + ESO	0.0202	2.9137	139.233	0.0009	96.478

**Table 7:** Performance Metrics of STSMC with ESO (Moderate Gains) and without ESO (High Gains).

Metric	STSMC + ESO (Moderate Gains)	STSMC (High Gains, No ESO)
RMS tracking error (rad)	0.00408	0.00576
Max tracking error (rad)	0.01893	0.02559
Saturation time ratio	0.0	0.0722
Chattering index RMS $\left(d\tau /dt\right)$ (N·m/s)	101.91	617.40

**Table 8:** Influence of ESO bandwidth parameter on tracking performance (elbow joint).

$\omega_{n}$	RMSE (e) (rad)	IAE (rad·s)	ITAE (rad·s^2^)	$R_{HF}$ (f ≥ 10 Hz)
5	0.0342	0.4721	5.102	0.0011
10	0.0296	0.3858	3.870	0.0014
15	0.0289	0.3795	3.761	0.0023

## Data Availability

The data that support the findings of this study are available from the corresponding author upon reasonable request.
